# Distal 2q duplication in a patient with intellectual disability

**DOI:** 10.1038/s41439-022-00215-8

**Published:** 2022-11-10

**Authors:** Toshifumi Suzuki, Hitoshi Osaka, Noriko Miyake, Atsushi Fujita, Yuri Uchiyama, Rie Seyama, Eriko Koshimizu, Satoko Miyatake, Takeshi Mizuguchi, Satoru Takeda, Naomichi Matsumoto

**Affiliations:** 1 Department of Obstetrics and Gynecology, Juntendo University Faculty of Medicine, Tokyo, 113-8421 Japan; 2 Department of Obstetrics and Gynecology, Keiai Hospital, Saitama, 354-0017 Japan; 3 Department of Pediatrics, Jichi Medical University, Shimotsuke, 329-0498 Japan; 4 Department of Human Genetics, Research Institute National Center for Global Health and Medicine, Tokyo, Japan; 5 Department of Human Genetics, Yokohama City University Graduate School of Medicine, Yokohama, 236-0004 Japan; 6 Department of Rare Disease Genomics, Yokohama City University Hospital, Yokohama, 236-0004 Japan; 7 Clinical Genetics Department, Yokohama City University Hospital, Yokohama, 236-0004 Japan; 8 Aiiku Research Institute for Maternal, Child Health and Welfare, Tokyo, Japan

**Keywords:** Clinical genetics, Development

## Abstract

We report on a patient with a distal 16.4-Mb duplication at 2q36.3-qter, who presented with severe intellectual disability, microcephaly, brachycephaly, prominent forehead, hypertelorism, prominent eyes, thin upper lip, and progenia. Copy number analysis using whole exome data detected a distal 2q duplication. This is the first report describing a distal 2q duplication at the molecular level.

## Introduction

Partial 2q duplication is a very rare chromosomal abnormality possibly arising from parental chromosomal rearrangements also involving a deletion of another partner chromosome. Therefore, pure distal 2q duplication without another partner chromosomal rearrangement is an extremely rare condition and its etiologies have not been fully understood^1–4^. Here, we describe a patient with a distal 16.4-Mb duplication and discuss the genetic and clinical aspects.

## Data report

The patient was the first child born normally to healthy non-consanguineous Japanese parents at 39 weeks of gestation. His family history was unremarkable. His birth weight was 2,708 g (21.2 centile). No fetal structural abnormality was detected by ultrasonography examination. At birth, no abnormality was pointed out. At the age of 10 months, the child was brought to our hospital because he could not sit alone. Brain magnetic resonance imaging showed a slightly small frontal lobe and brachycephaly, whereas myelinization of the nervous tissue was normal (Supplementary Fig. S1). He showed microcephaly of 44.2 cm (10th centile) at 14 months, brachycephaly, prominent forehead, hypertelorism, prominent eyes, thin upper lip, and progenia (Table 1). He showed normal developmental milestones, but he did not speak single words at 6 years. G-banded chromosome analysis of the patient and his parents was reported as normal.

**Table 1:** Clinical features of the patients with distal 2q duplication.

	This case	Fritz et al.	Elbracht et al.	Hermsen et al.	Dahoun-Hadorn et al.		
Location of duplication	2q36.3-2qter	2q35-2q37.1	2q35-2q37.3	2q35-2q37.3	2q35-2qter	Frequency	(%)
Race	Japanese	Croatian	Russian	NA	NA	–	–
Age at report (years)	6	7	16	9	7	–	–
Sex	Male	Male	Female	Female	Male	–	–
Gestation(weeks)	Delivery at term	Delivery at term	Delivery at term	Delivery at term	Delivery at term	–	–
Body size at birth							
Weight (g)	2,708 (21.2 centile)	3,200 (50th centile)	3,210 (25-50th centile)	NA	4,000 (90th centile)	–	–
Body heigh (cm)	NA	51 (75th centile)	56 (97th centile)	NA	NA	–	–
Occipito-frontal circumference (cm)	NA	NA	34 (25th centile)	NA	NA	–	–
Body size at report							
Weight (kg)	9.7 (25th centile, 19 months)	19.7 (10th centile)	NA	24.6 (10th centile)	26 (75-90th centile)	–	–
Body heigh (cm)	79.5 (25th centile, 19 months)	120 (25th centile)	177.6 (97th centile)	131.6 (35th centile)	124 (50th centile)	–	–
Occipito-frontal circumference (cm)	44.2 (10th centile, 14 months)	49.9 (10-25 centile)	56.4 (97th centile)	50 (8th centile)	56 (> 97th centile)	–	–
Growth retardation	–	+	–	+	–	2/5	40
Intellectual disability	+ (Severe)	+	+ (Moderate)	+ (Moderate)	+ (Limited to a dozen words at 7 years)	5/5	100
Microcephaly	+	–	–	NA	–	1/4	25
Cerebral atrophy	- (Slightly small frontal lobe)	NA	NA	NA	–	0/2	0
Brachycephaly	+	+	+	NA	–	3/4	75
Abnormality of the hairline	–	+	+	NA	–	2/4	50
Prominent forehead	+	+	+	+	+	5/5	100
Broad nasal bridge	–	+	+	+	+	3/5	60
Overhanging nasal tip	–	+	+	+	+	4/5	80
Hypertelorism	+	–	–	+	+	3/5	60
Ocular anomalies	+ (Prominent eyes)	–	+	+ (Nystagmus and slow signal conduction of the optical nerve)	–	3/5	60
Long philitrum	–	–	–	+	–	1/5	20
Thin upper lip	+	+	+	+	+	5/5	100
High arched/cleft palate	–	+	–	–	–	1/5	20
Micro retrognathia	–	NA	+	NA	+	1/3	33.3
Dental abnormalities	+ (Progenia)	+	NA	+	NA	3/3	100
Hearing disorders	–	–	–	–	–	0/0	0
Large ears	–	+	+	+	+	4/5	80
Low set ears	–	+	–	+	+	3/5	60
Short neck	–	+	NA	NA	NA	1/2	50
Clinodactyly of fifth ginger	–	+	+	+	+	4/5	80
Skeletalanomalies	–	Short with incomplete syndactyly III/IV of both hands and II/III of both feet	–	–	Big and ling toes with enlarged distal phalanges Abnormally-placed sphenoid wings Small suprasellar calcification and a large temporal fossa	2/5	40
Cardiac anomalies	–	NA	–	–	–	0/4	0
Genital anomalies	–	+	+	–	+	3/5	60
Urinary organ anomalies	–	NA	–	–	+	1/4	25
Muscular hypotonia	–	+	NA	NA	–	1/3	33.3
Others	–	Joints with hyperextensible	–	–	Clacification of the optic chiasma cistern	2/5	40

*NA* not available.

The institutional review board of the Yokohama City University School of Medicine approved this study. Peripheral blood samples were collected from the patient and his parents after obtaining written informed consent. Genomic DNA extracted from blood leukocytes was used for the genetic analysis. Whole exome sequencing (WES) of the extracted DNA samples was performed on a HiSeq 2500 platform (Illumina, San Diego, CA, USA) with 101 bp paired-end reads. After quality control, the reads were aligned to the human reference genome (UCSC hg19, NCBI build 37.1, https://genome.ucsc.edu/) using NovoAlign (http://www.novocraft.com/products/novoalign/). The WES analysis procedure was as described previously^5–7^. Possible pathogenic variants were evaluated using SIFT (http://sift.jcvi.org/), PolyPhen2 (http://genetics.bwh.harvard.edu/pph2/), and MutationTaster (http://MutationTaster.org/). Possible pathogenic variants were confirmed by Sanger sequencing. Copy number variations (CNVs) were analyzed using the WES data with a slightly modified eXome Hidden Markov Model (XHMM) and Nord’s program based on relative depth of coverage ratios^8,9^. Candidate CNVs were validated by quantitative PCR (qPCR) at the selected spots as described previously^5^. Normalization was performed with an autosomal internal control locus (*STXBP1* and/or *FBN1*). CNVs were compared among an unrelated control individual, the patient, and his parents. The PCR conditions and primer sequences are available on request.

**Fig. 1:**
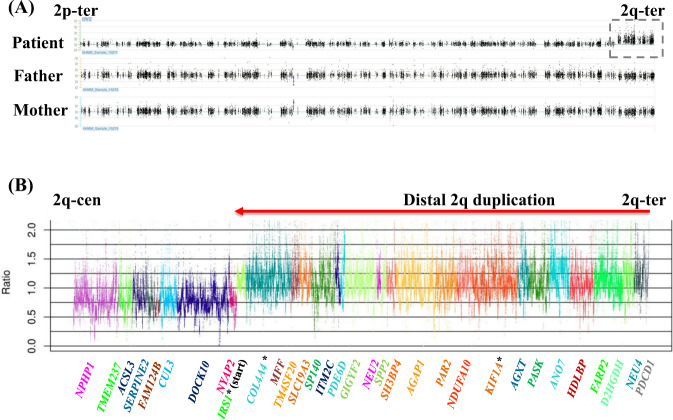
Copy number analysis on chromosome 2 using whole exome sequencing data. **A** Distal 2q duplication detected by an eXome Hidden Markov Model. The dashed-line box indicates the distal 2p duplication. **B** Copy number variations analysis by Nord’s method. The red arrow indicates the duplicated region. Asterisks indicate the genes used for qPCR. The 134 protein cording genes in the duplicated region are listed in Supplementary Table S1.

Firstly, we performed WES of the patient’s DNA sample, but no pathogenic single-nucleotide variants (SNVs) were detected. The CNVs analysis using XHMM also did not detect any pathological CNVs. Subsequently, trio-based WES and CNVs analysis were performed using trio-WES data and XHMM, and a distal 2q duplication of 16.4-Mb was detected of the patient’s DNA sample (Fig. 1A). We analyzed the duplicated region using Nord’s method focusing on candidate genes within the 16.4-Mb region, and confirmed the duplication from *IRS1* to *RTP5* at 2q36.3-qter, which contained 134 protein coding genes (Fig. 1B and Supplementary Table S1). The qPCR analysis confirmed the duplication at *IRS1*, *COL4A4*, and *KIF1A* (Supplementary Fig. S2). We re-examined the result of G-banded chromosome analysis in the patient. However, we could not recognize a distal 2q duplication. This duplication has not been registered in the ClinGen database (https://www.clinicalgenome.org/) or the Database of Genomic Variants (http://dgv.tcag.ca/dgv/app/home) of normal controls.

We counted the paternal and maternal alleles aided by SNVs in the duplicated region (Supplementary Table S2). In the patient, maternal reads were counted as approximately twice the paternal reads, indicating that maternal duplication had occurred (Supplementary Table S2).

## Discussion

In a previous study, 2q duplications were found to arise mostly from a parental chromosomal rearrangement associated with the deletion of a partner chromosomal segment^10^. Pure partial 2q duplication with no deletion of a different partner chromosomal segment is extremely rare. To our knowledge, only four cases with pure 2q35-qter at G-banded chromosome level have been reported^1–4^, but without any precise information of duplication regions at the molecular level.

In the four previous reported cases and in our patient, intellectual disability (5/5, 100%), brachycephaly (3/4, 75%), prominent forehead (5/5, 100%), broad nasal bridge (3/5, 60%), overhanging nasal tip (4/5, 80%), hypertelorism (3/5, 60%), ocular anomalies (3/5, 60%), thin upper lip (5/5, 100%), dental abnormalities (3/3, 100%), large ears (4/5, 80%), low set ears (3/5, 60%), clinodactyly of fifth finger (4/5, 80%), and genital anomalies (3/5, 60%) were relatively common (Table 1), suggesting these characteristics may be important clinical features of distal 2q duplication. Interestingly, in two male patients reported previously, genital abnormalities were recognized including shawl scrotum, hypospadias, and cryptorchidism^1,3^, but the case reported here had no genital anomaly (Table 1). The four previously reported cases were analyzed only at the chromosomal (not molecular) level, so it is possible that the inconsistent clinical features may be attributed to imprecise duplicated regions among them.

Our patient had an approximately 16.4-Mb 2q duplication at 2q36.3-37.3, a region that included at least 134 protein cording genes. Among them, variants in 26 genes are known to lead to Mendelian diseases, but no triplosensitivity (TS) gene (TS score of 2 or 3) was found, implying there was no strong contribution of a single gene in the distal 2q duplication (Supplementary Table S1).

WES data suggested that a duplication in this patient derived from maternal allele. In six heterozygous SNVs mapped to the duplicated region in the patient and mother, all maternally derived reads were counted as twice comparing the wild-type reads in the patient (Supplementary Table S2). If the duplication was derived from two maternal homologous chromosomes, variant reads should be half of wild-type reads in count. Therefore, these results indicated that its maternal duplication was derived from only a single homologous chromosome 2 in the mother. We could not determine whether the duplication is direct (tandem) or inverted or insertion of another chromosome.

In summary, we have described a distal 2q duplication in a patient at the molecular level for the first time. However, further evidence for its etiology is needed.

## Supplementary information

supplementary information

## Data Availability

The relevant data from this Data Report are hosted at the Human Genome Variation Database at 10.6084/m9.figshare.hgv.3240.
