# Supplementary material for: Distal 2q duplication in a patient with intellectual disability
Source: Hum Genome Var. 2022 Nov 10;9:39. doi: 10.1038/s41439-022-00215-8 (PMC9649592; doi:10.1038/s41439-022-00215-8)

**Supplementary information**

**Distal 2q duplication in a patient with** **intellectual disability**

Toshifumi Suzuki, Hitoshi Osaka, Noriko Miyake, Atsushi Fujita, Yuri Uchiyama, Rie Seyama, Eriko Koshimizu, Satoko Miyatake, Takeshi Mizuguchi, Satoru Takeda, and Naomichi Matsumoto

**Correspondence to:**

Naomichi Matsumoto

Department of Human Genetics, Yokohama City University Graduate School of Medicine, Yokohama 236-0004, Japan

Tel: +81-45-787-2606

Fax: +81-45-786-5219

E-mail: naomat@yokohama-cu.ac.jp (N. Matsumoto)

**Supplementary information**

**・Supplementary Tables S1 and S2**

**・Supplementary Figs. S1 and S2**

**Supplementary Table S1. RefSeq protein cording genes in the distal 2q duplicated region.**

**Supplementary Table S2. Allele ratios of parental-derived single-nucleotide variants in the patient**

**Supplementary Fig. S1 Magnetic resonance images of the patient’s brain at the age of 10 months.**

1. T2-weighted axial image showing slightly small frontal lobe. (B) T2-weighted sagittal image showing slightly small frontal lobe and brachycephaly.

**
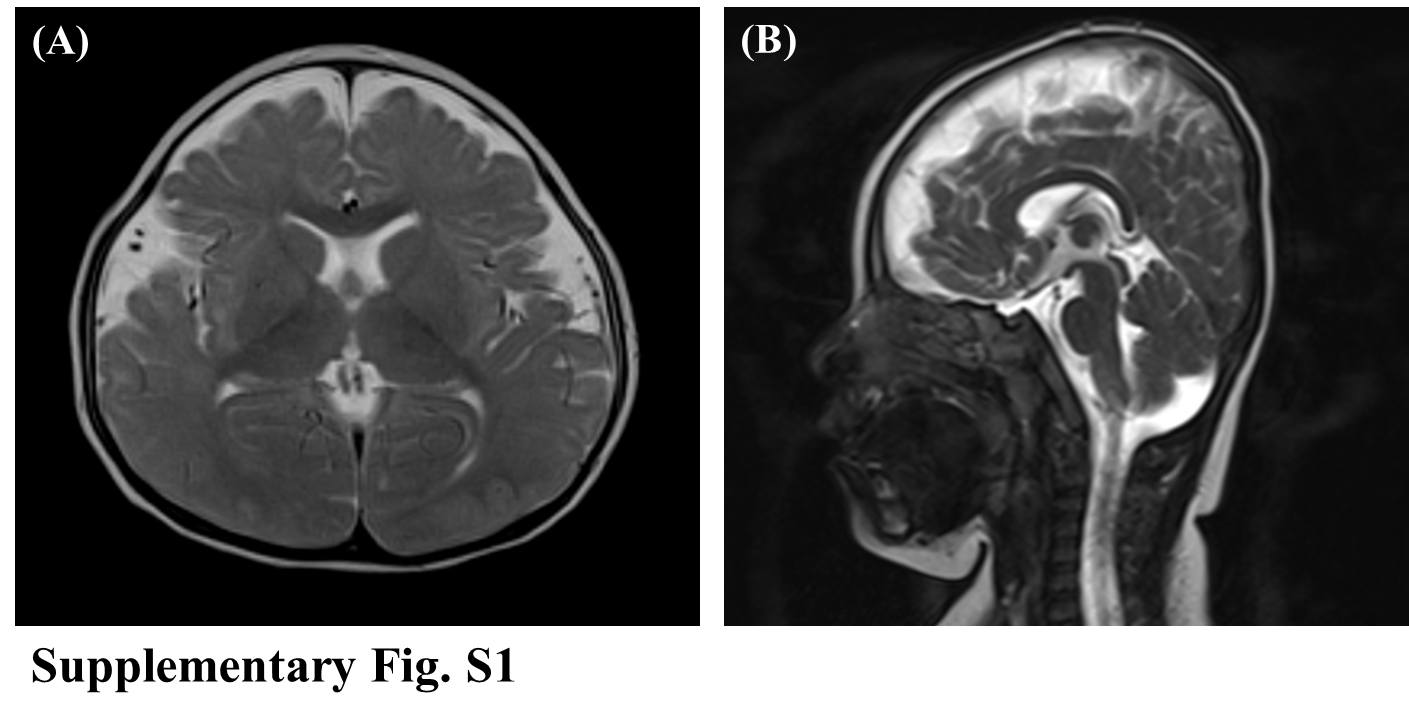
**

**Supplementary Fig. S2 Confirmation of the distal 2q duplication by qPCR.**

The patient’s sample was compared with those of an unrelated normal individual and his parents at distal 2q loci corresponding to *IRS1*, *COL4A4*, and *KIF1A*.


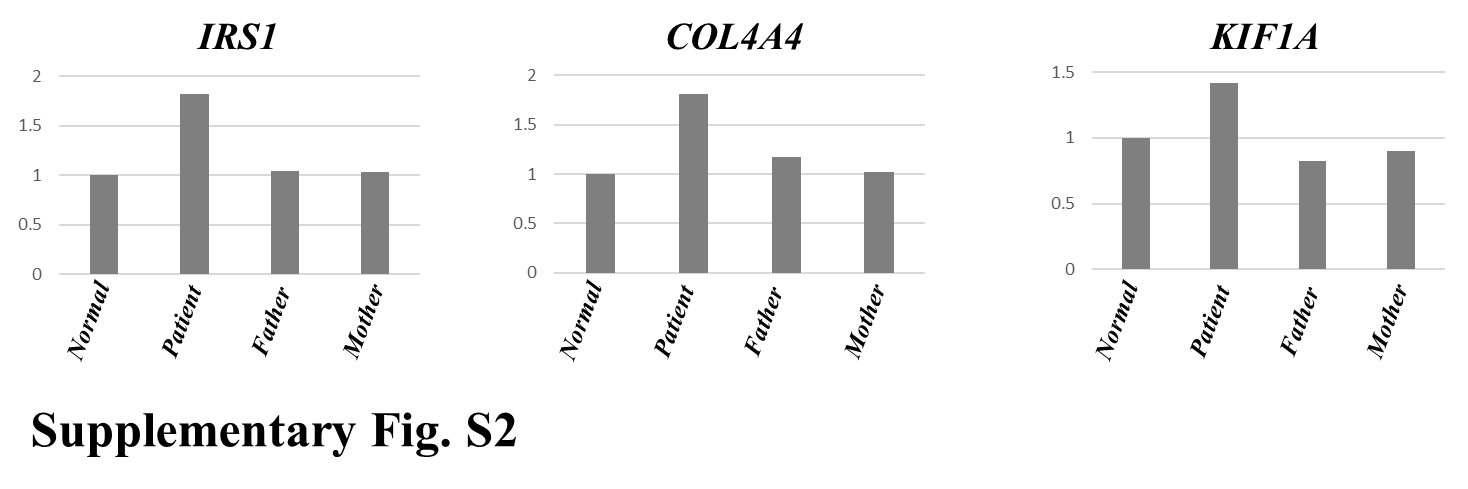

Supplement: Supplementary file 1 — supplementary information [file 41439_2022_215_MOESM1_ESM.docx]
